# Prenatal Care Disruptions and Associations With Maternal Mental Health During the COVID-19 Pandemic

**DOI:** 10.3389/fgwh.2021.648428

**Published:** 2021-04-23

**Authors:** Taylor Groulx, Mercedes Bagshawe, Gerald Giesbrecht, Lianne Tomfohr-Madsen, Erin Hetherington, Catherine A. Lebel

**Affiliations:** ^1^Alberta Children's Hospital Research Institute, University of Calgary, Calgary, AB, Canada; ^2^Department of Radiology, University of Calgary, Calgary, AB, Canada; ^3^Hotchkiss Brain Institute, University of Calgary, Calgary, AB, Canada; ^4^Department of Psychology, University of Calgary, Calgary, AB, Canada; ^5^Department of Pediatrics, University of Calgary, Calgary, AB, Canada; ^6^Department of Obstetrics and Gynaecology, University of Calgary, Calgary, AB, Canada

**Keywords:** pregnancy, prenatal care, pandemic, depression, anxiety, COVID-19, stress

## Abstract

As the novel coronavirus (COVID-19) spread across Canada in March 2020, provinces imposed restrictions. These changes impacted how pregnant individuals received prenatal care and experienced childbirth. The stress caused by these changes may negatively affect the well-being of pregnant individuals with impacts on the developing child. This study investigated the impact of the pandemic on prenatal care and birth plans of pregnant individuals in Canada and potential associations with maternal mental health. Data from 4,604 participants was collected from English- and French-speaking Canadians between April 5 and June 1, 2020 as part of the Canada-wide Pregnancy During the COVID-19 Pandemic study. Symptoms of maternal depression, general anxiety, and pregnancy-related anxiety were assessed. Participants also answered questions about disruptions and changes to prenatal care and their birth plans due to the COVID-19 pandemic. Logistic regression was used to estimate associations between prenatal care disruptions and maternal mental health. Cancellation of prenatal appointments and birth plan changes (specifically changes to childcare during birth and change of support person attending the birth) were significantly associated with greater odds of experiencing clinically elevated depression, anxiety, and/or pregnancy-related anxiety symptoms. These results highlight the need for reliable and accessible prenatal care during the pandemic, such as the integration of mental health screenings and co-ordination of prenatal care providers.

## Introduction

Proper prenatal care is important for the health of both the pregnant individual and the developing baby. Inadequate prenatal care has been associated with low birth weight, preterm birth, and miscarriage (1–5). The emergence of the novel coronavirus (COVID-19) led to widespread restrictions that disrupted prenatal care for many pregnant individuals around the world. During the COVID-19 pandemic, the Canadian government recommended remote (virtual) appointments with doctors and obstetricians where possible (6), and some hospitals and healthcare providers prevented support persons from prenatal appointments, ultrasounds, or the birthing room, switched to virtual appointments, and/or limited in-person meetings with care teams. Restrictions and changes to prenatal care and birth protocols occurred quickly, adding potential uncertainty and stress for pregnant individuals.

Prior studies show that 10–25% of individuals experience mild to moderate anxiety and/or depression during pregnancy (7). Prenatal depression and anxiety have been linked to a negative perception of the birth experience (8), greater risk of postnatal depression (9–12), and loss of interest in the child (13). Prenatal stress is also related to adverse cognitive and behavioral outcomes in children (14–17). Pregnancy-related anxiety refers to worries or fears in relation to childbirth, the safety and the health of the baby, and future parenting. Higher pregnancy-related anxiety is strongly associated with adverse birth outcomes, including preterm birth and low birth weight, problematic infant temperament, behavioral and emotional problems in the child, and developmental delays (18–20). Recent studies indicate substantially elevated symptoms of anxiety and/or depression in pregnant individuals during the current COVID-19 pandemic compared to pre-pandemic pregnancy cohorts (21–25).

The goal of the present study was to determine the impact of the COVID-19 pandemic on prenatal care and birth plans of pregnant individuals in Canada and how these changes were associated with maternal mental health.

## Materials and Methods

### Participants

The current study reports data collected from the Pregnancy during the COVID-19 Pandemic study (26) between April 5 and June 1, 2020. These dates were chosen to capture the effects of the initial lockdown period. The ongoing Pregnancy during the COVID-19 Pandemic study recruited pregnant individuals across Canada using social media, primarily via Facebook and Instagram ads, to complete an online survey. The inclusion criteria were as follows: living in Canada, able to read and write English and/or French, 17 years of age or older, and having a confirmed pregnancy <35 weeks' gestation. This study was approved by the University of Calgary Conjoint Health Research Ethics Board (REB20-0500).

### COVID-19 and Prenatal Care

Participants completed a questionnaire about disruptions and changes to their prenatal care and birth plan due to the current COVID-19 pandemic. Participants were presented with the following questions/statements: “Have you experienced changes in the way that prenatal care is delivered to you during the COVID-19 pandemic?,” “Have any of your prenatal care appointments been canceled?,” and “Are you able to bring your partner or support person to your appointments?” Participants answered these questions using a binomial scale (yes/no). Participants were also asked “Which changes have you made to your birth plan (check all that apply): Birth Location; Support People; Childcare Arrangements; Other changes”.

### Anxiety and Depression Symptoms

The Edinburgh Postnatal Depression Scale (EPDS) (27, 28) was used to measure maternal depressive symptoms. Although the EPDS is not diagnostic, scores ≥13 have been shown to have maximal consistency with a diagnosis of major depressive disorder and are used to identify individuals with clinically concerning depression symptoms (27). Using a cut-off of 13 for the EPDS, sensitivity ranges from 38 to 43% (trimester dependent) and specificity is 98–99% (29). The PROMIS Anxiety Adult 7-item short form was used to assess general anxiety. Scores ≥60 on this measure have been associated with clinically elevated anxiety, possible scores range from 36.3 to 82.7 (30). Data from the EPDS and PROMIS measures were dichotomized at the established cut-off scores, ≥13 and ≥60 respectively, representing clinically elevated symptoms. Official French translations were used for each measure. Pregnancy-related anxiety, referring to fear and worries surrounding the circumstances of birth and health of baby, was assessed differently in the English and French sample due to availability of measures. Responses to the English survey were assessed using a 10-item questionnaire (PRAQ), in which participants pick from four possible responses. The French survey used a similar, but slightly different, validated 10-item questionnaire (PRAQ-R2), in which participants chose from five responses per question (31). Neither the PRAQ (English) nor the PRAQ-R2 (French) provide cut-off scores for clinically elevated symptoms; previous treatment studies commonly use a median split method to define groups with higher vs. lower pregnancy related symptoms (32). We used a conservative approach and used the upper quartile to define individuals with elevated symptoms (PRAQ ≥ 24 and PRAQ_R2 ≥ 30).

### Statistical Analysis

IBM SPSS Statistics 26 was used for all statistical analysis. Survey responses were manually checked for accuracy before analysis, and invalid records (e.g., implausible due date) were removed. The associations between clinically elevated psychological symptoms and COVID-19 related disruptions to prenatal care and birth plans were estimated using multivariable logistic regression using IBM SPSS. Separate logistic regressions were completed for each measure of mental health in relation to prenatal care disruptions and changes to birth plans. The PROMIS Anxiety and EPDS measures were analyzed from English and French survey responses in combination. Analysis for the PRAQ and PRAQ-R2 were completed separately for participants completing the survey in English or French, as had different score ranges. Logistic regression models were adjusted for age, parity, ethnicity, trimester, maternal education, and household income as covariates. A supplementary analysis was conducted controlling for prior history of anxiety and depression, in addition to other covariates (age, parity, trimester, income, maternal education, ethnicity). Missing data was coded as N/A and those values did not contribute to the statistical model.

## Results

### Participants

A total of 4,604 records collected between April 5 and June 1, 2020 were included in the current analysis. 3,755 participants completed the survey in English and 849 in French. Participants were aged 31.56 ± 4.39 years (range 18–49 years). The majority of participants were married (67.0%) or living with a partner (28.6%). All participants lived in Canada, with the majority residing in Ontario (28.3%), Alberta (23.2%), and Quebec (21.0%). Most participants self-identified as Caucasian (81.6%), with others identifying as First Nations (1.0%), Metis (1.2%), Black (1.7%), Chinese (1.7%), Filipino (1.4%), Korean (0.2%), West Asian (0.6%), South Asian (3.3%), South-east Asian (0.3%), Hispanic (2.1%), and Mixed (4.9%).

Participants reported their highest level of education, having completed at least a bachelor's degree (40.3%), a trade or community college diploma (24.0%), or a master's degree (18.6%). Participants had a median income range of CAD $100,000–124,999 per year (USD $75,000–95,000). 45.6% of participants reported having other children (32.9% had one child, 9.6% had two children, and 3.2% had three or more children). Average gestation of participants when they completed the survey was 21.6 ± 8.5 weeks (range 3.7–35). Prior history of mental health conditions was reported; 39.7% of participants had a history of anxiety and 18.8% had a history of depression.

### Prevalence of Mental Health Symptoms

**Figure 1:**
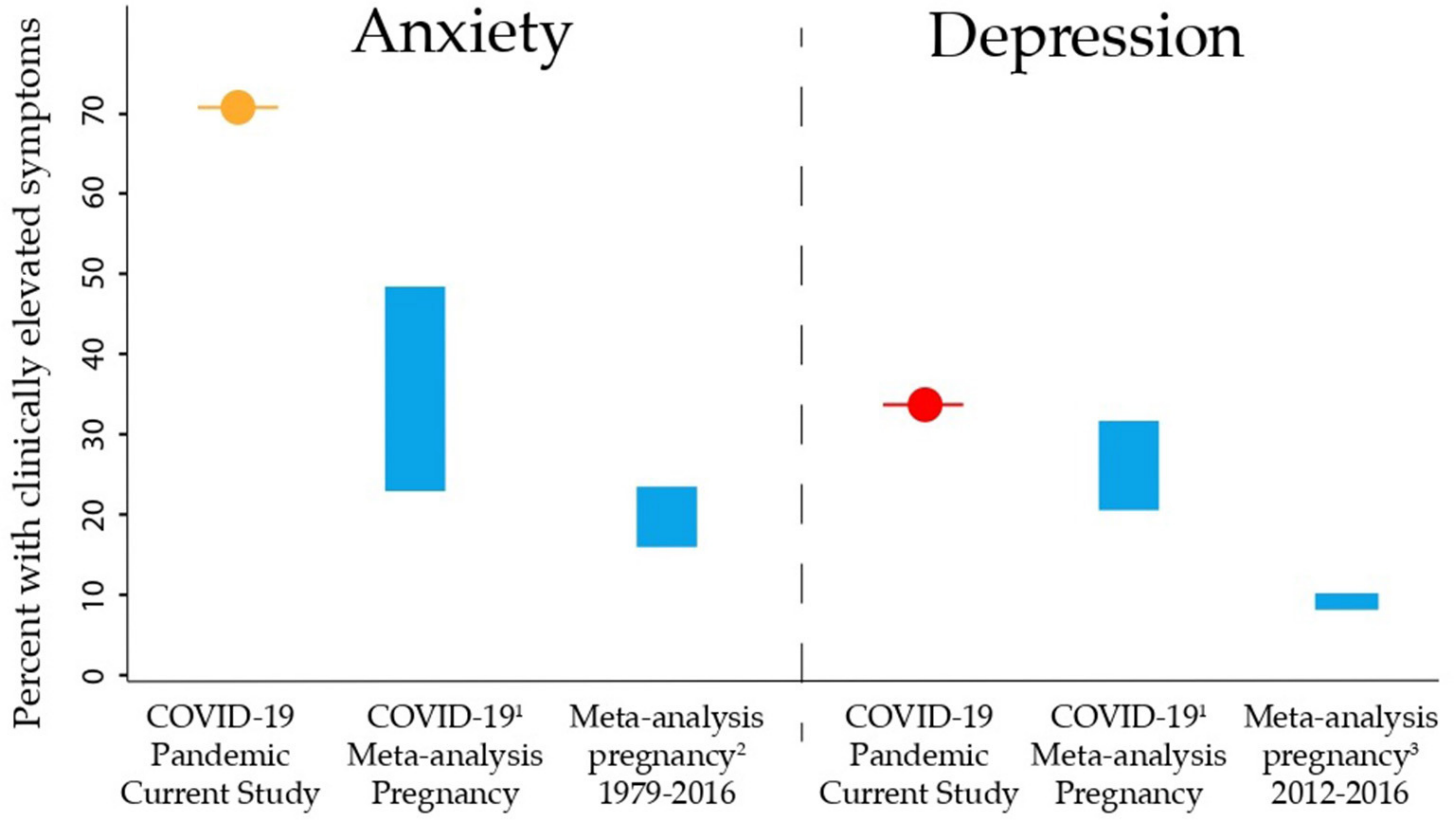
Rates of clinically elevated anxiety and depression symptoms in pregnant individuals in this study were compared to pre-pandemic meta-analysis estimates, and meta-analysis estimates of rates during the COVID-19 pandemic. The prevalence in the current study is given, vs. the estimated ranges in prior studies. The frequency of clinically elevated symptoms of anxiety (orange) and depression (red) in the current study were substantially higher than pre-pandemic and during-pandemic estimates, likely because this data was collected early in the COVID-19 pandemic (April–June 2020) when uncertainty was at its highest. References 1, Tomfohr-Madsen et al. (33); 2, Dennis et al. (34); 3, Gavin et al. (35).

Mean scores on mental health measures can be found in Table 1. 34.0% of participants experienced clinically elevated symptoms of depression (EPDS scores ≥13), and 70.9% of participants had clinically elevated symptoms of anxiety (PROMIS scores ≥60) (Figure 1).

**Table 1:** Demographics, mental health scores, and disruptions to prenatal care.

**Measure**	***n***	**Mean**	**Standard deviation**	**Range**
Gestation (weeks)	4,604	21.63	8.48	3.7–35
Age (years)	4,604	31.56	4.39	18–49
**Mental health scores**				
Edinburgh postnatal depression scale (EPDS)	4,491	10.30	5.37	0–30
PROMIS anxiety T-scores	4,477	58.74	8.2	36–82.7
Pregnancy-related anxiety questionnaire (PRAQ)				
English PRAQ measure	3,681	21.23	5.26	8–40
French PRAQ-R2 measure	789	25.71	7.84	10–48
	***N***	**%**		
**Disruptions to prenatal care**				
Changes in way prenatal care had been delivered	4,115/4,604	89.4% Yes		
Cancellation of prenatal care appointments	1,644/4,114	40% Yes		
Support person allowed to attend prenatal appointments	4,21/4,604	9.1% Yes		
Changes to birth plan	1,535/4,604	33.3% Yes		
- Birth location	434/4,599	9.4% Yes		
- Support person	1,229/4,599	26.7% Yes		
- Childcare plan	499/4,599	10.8% Yes		
- “Other” changes	204/4,599	4.4% Yes		

### Disruptions and Changes to Prenatal Care

Eighty-nine percent of participants reported changes in the way that prenatal care was delivered to them during the COVID-19 pandemic (Table 1). Forty percent of respondents reported the cancellation of at least one prenatal care appointment and 90.9% of participants were not permitted to bring their partner or support person to prenatal appointments. 33.4% of participants made changes to their birth plan because of the COVID-19 pandemic: 9.4% changed their birth location, 26.7% changed their birth support person(s), and 10.9% changed their childcare plan (some participants made changes to multiple factors and thus numbers do not add to 33.4%).

### Associations Between Prenatal Care Disruptions and Mental Health

**Figure 2:**
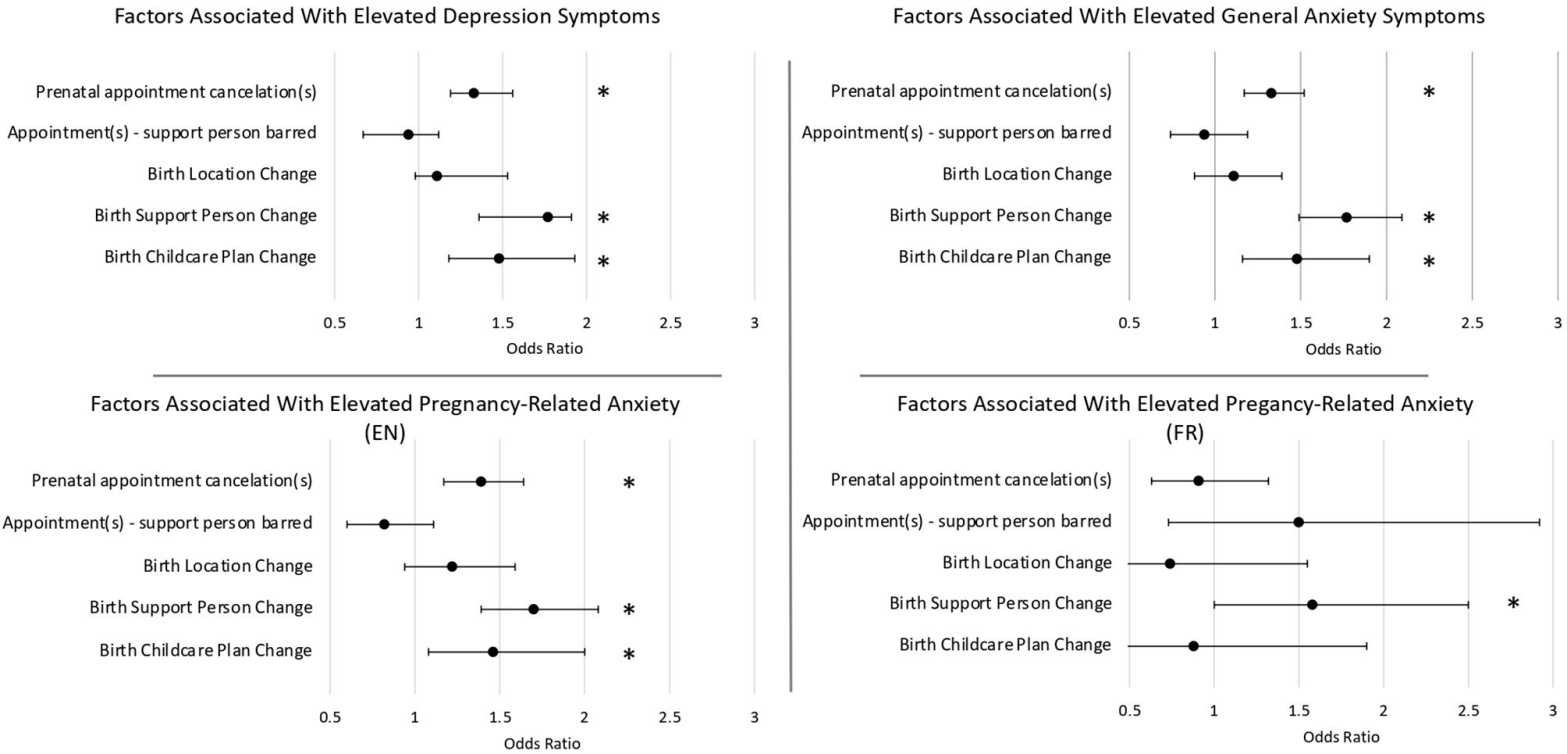
These odds ratios demonstrate how each of the COVID-related disruptions to prenatal care affects the odds of experiencing elevated symptoms of psychological distress. Odds ratios were adjusted for age, parity, ethnicity, trimester, maternal education, and household income. Note that the sample size for the French Pregnancy-Related Anxiety Questionnaire (PRAQ-R2) was smaller than for the English PRAQ. *Indicates statistical significance (*p* < 0.05).

Cancellation of prenatal appointments was associated with increased odds of experiencing clinically elevated depression symptoms (*OR* = 1.36, 95% *CI* [1.19, 1.56], *p* < 0.001), and clinically elevated general anxiety (*OR* = 1.33, 95% *CI* [1.17, 1.52], *p* < 0.001) (see Table 2, Figure 2). Changes to the planned support person(s) attending the birth increased odds of clinically elevated depressive symptoms (*OR* = 1.61, 95% *CI* [1.36, 1.91], *p* < 0.001) and general anxiety symptoms (*OR* = 1.77, 95% *CI* [1.49, 2.09], *p* < 0.001). Changes to planned childcare arrangements during labor also increased the odds of clinically elevated depressive symptoms (*OR* = 1.50, 95% *CI* [1.18, 1.92], *p* = 0.001) and anxiety symptoms (*OR* = 1.48, 95% *CI* [1.16, 1.90], *p* = 0.002). Among English responses, odds of high pregnancy-related anxiety were increased by cancelation of prenatal appointments (OR=1.39, 95% *CI* [1.17, 1.64], *p* < 0.001), changes to planned support person(s) (OR=1.70, 95% *CI* [1.39, 2.08], *p* < 0.001), and changes to childcare during labor (*OR* = 1.46, 95% *CI* [1.08, 2.00], *p* = 0.015). Within French responses, only changes to the planned support person(s) able to attend the birth were associated with increased odds of pregnancy-related anxiety symptoms (*OR* = 1.58, 95% *CI* [1.00, 2.50], *p* = 0.049), though this smaller sample has reduced power.

**Table 2:** Prenatal care disruptions and symptoms of maternal distress^a^.

	**Odds ratio**	**95% CI for odds ratio**		***p*-Value**
		**Lower**	**Upper**	
**General anxiety symptoms**				
Prenatal appointment canceled	**1.33**	1.17	1.52	**0.000**
Support person(s) not allowed to attend prenatal appointments	0.94	0.74	1.19	0.61
Birth location change	1.11	0.88	1.39	0.38
Birth support person change	**1.77**	1.49	2.09	**0.000**
Birth childcare changes	**1.48**	1.16	1.90	**0.002**
**Depression symptoms**				
Prenatal appointment canceled	**1.36**	1.19	1.56	**0.000**
Support person(s) not allowed to attend prenatal appointments	0.87	0.67	1.12	0.280
Birth location change	1.23	0.98	1.53	0.080
Birth support person change	**1.61**	1.36	1.91	**0.000**
Birth childcare changes	**1.50**	1.18	1.92	**0.001**
**Pregnancy-related anxiety symptoms (english measure)**				
Prenatal appointment canceled	**1.39**	1.17	1.64	**0.000**
Support person(s) not allowed to attend prenatal appointments	0.815	0.60	1.11	0.190
Birth location change	1.22	0.94	1.59	0.130
Birth support person change	**1.70**	1.39	2.08	**0.000**
Birth childcare changes	**1.46**	1.08	2.00	**0.015**
**Pregnancy-related anxiety symptoms (french measure)**				
Prenatal appointment canceled	0.91	0.63	1.32	0.628
Support person(s) not allowed to attend prenatal appointments	1.50	0.73	2.92	0.284
Birth location change	0.74	0.35	1.55	0.422
Birth support person change	**1.58**	1.00	2.50	**0.049**
Birth childcare changes	0.88	0.41	1.90	0.746

a *Model adjusted for maternal education, age, ethnicity, household income, parity, and trimester. Bold values indicate significance at p < 0.05*.

A supplementary analysis was conducted controlling for prior history of anxiety and depression, in addition to other covariates. All relationships between mental health and changes to prenatal care and birth plans remained significant, except the association between pregnancy related anxiety in the French sample and changes to birth support person (*OR* = 1.51, 95% *CI* [0.93, 2.42], *p* = 0.086).

## Discussion

Here, we show that prenatal care disruptions were common during the COVID-19 pandemic, and were associated with clinically elevated depression, anxiety, and pregnancy-related anxiety symptoms. Eighty nine percent of pregnant individuals in this study reported at least one pandemic-related change in their prenatal care. Of these changes, the most common were inability to have support persons attend prenatal appointments (90.6% of participants) and cancellations of prenatal care appointments (40% of participants). COVID-19-related changes in prenatal care may contribute to uncertainty around maternal and fetal health, which may compound the already elevated levels of stress, further increasing the risk of psychological distress. Care providers should work to provide pregnant individuals with consistent and informed prenatal care in an effort to reduce uncertainty and stress. Stress during pregnancy increases the risk of physical and psychological problems in both the pregnant individual and the child (36–38). Thus, these care changes and related increases to anxiety and depression symptoms, have concerning potential long-term implications for children. Given the benefits of support during pregnancy (37, 39), when considered in conjunction with the elevated psychological distress in this population, the need for consistent and supportive care from medical professionals and the prenatal care team should be considered of utmost importance.

Cancellations of prenatal appointment(s), changes to the planned support person(s) attending the birth and changes to childcare plans during labor are all associated with elevated pregnancy-related anxiety in the English respondents, while only a change in support person(s) attending the birth was associated with elevated pregnancy-related anxiety in the French subset. These distinctions between the English and French subsamples potentially reflect the differences in questionnaires used, the smaller sample size of French-speaking participants (which reduces power), and/or differences in how prenatal care was delivered or perceived in Quebec (where most French respondents lived) compared to the rest of the country. Care should be taken to ensure an individualized approach to prenatal care during the COVID-19 pandemic to mitigate pregnancy-related anxiety in a manner which is specific to the individual in question.

Due to the COVID-19 pandemic, many forms of medical care have transitioned to telehealth (40). Remote delivery of prenatal care without the face-to-face experience has the potential to be perceived as less supportive, despite being an adequate substitute for complete cancellation (40, 41). When prenatal appointments were in person, most participants in this study were not allowed to bring their partner or support person, which may also contribute to a perceived decrease in support. Decreased social support is associated with higher symptoms of anxiety and depression. A result of note was that not being allowed to bring one's support person(s) to prenatal appointments was not associated with maternal psychological distress in this study. With video- or telephone-based remote appointments available for prenatal appointments when they are canceled, individuals can attend these appointments from the comfort of their own home. This may result in an increased level of comfort and perception of support contributing to this result, as compared to when the individual must travel for their appointments. We do not have data regarding the replacement of prenatal appointments with remote appointments, thus this cannot be concluded from these results; further research is needed to expand on this possibility.

Our sample includes pregnant individuals from across Canada. 40.3% of our sample held at least a Bachelor's degree, which is similar to 2016 Canadian Census data showing that 40.7% of women aged 25–34 held at least a Bachelor's degree (42). Statistics Canada reports that 22.3% of Canada's total population is a visible minority, which is slightly higher than the proportion in our sample (18.4%) (42). Clinically elevated symptoms of anxiety were reported by 70.9% of participants, and clinically elevated symptoms of depression by 34% of participants in the current study. These estimates are higher than a recent meta-analysis of rates during the COVID-19 pandemic, and higher than pre-pandemic meta-analyses indicate (see Figure 1) (33–35). The higher rate of psychological symptoms reported here may be due to the time period of this study, which collected data early in the pandemic (April–June 2020), when uncertainty was very high, and restrictions were most severe. The proportion of participants in this sample with a prior history of anxiety (39.7%) is higher than would be expected (8.7–11.6%) (43, 44). This could be an attributing factor as to why clinically elevated anxiety symptoms for the sample are much higher (70.9%) than pre-pandemic meta-analysis estimates and estimates of rates during the COVID-19 pandemic (see Figure 1).

This study has some potential limitations that should be considered when interpreting the results. The primary recruitment method for the Pregnancy During the Pandemic study was advertising on social media, which may have led to a selection bias. Additionally, this study employed the use of screening tools for psychological distress as opposed to diagnostic tools. While these screening tools were previously validated and correlate with diagnostic outcomes (27–31), future studies may look to reinforce these findings with clinical diagnosis. Results may not be generalizable to the entire population, and future studies may consider recruiting participants through alternate methods to capture a different sample.

Pregnant individuals are experiencing significant psychological distress during the COVID-19 pandemic (21–25). Our research provides new insight into the relationship between disruptions to prenatal care and this distress. Prenatal care disruptions were common, and appointment cancellations as well as changes to the birth plan were associated with clinically elevated depression and/or anxiety symptoms. This study illustrates the need for reliable and accessible prenatal care during the current pandemic, including integration of mental health screenings and modified birth plans that create a supportive birthing environment and are person-centered. In order to maintain high quality of care in the face of a public health crisis, prenatal, and medical care professionals should ensure that they are delivering high-quality care in a personal way that maximizes support felt by the individual, which may require additional training in remote methods of care delivery. Care providers should work together to provide pregnant individuals with consistent prenatal care to reduce uncertainty.

## Data Availability Statement

The raw data supporting the conclusions of this article will be made available by the authors, without undue reservation.

## Ethics Statement

The studies involving human participants were reviewed and approved by This study was approved by the University of Calgary Conjoint Health Research Ethics Board (REB20-0500). Written informed consent from the participants' legal guardian/next of kin was not required to participate in this study in accordance with the national legislation and the institutional requirements.

## Author Contributions

GG, LT-M, and CL conceptualized the larger Pregnancy during the COVID-19 Pandemic study. TG and MB led statistical analysis and wrote the original manuscript draft. All authors contributed to the article, design of this analysis, critical revisions of the manuscript, and approved the submitted version.

## Conflict of Interest

The authors declare that the research was conducted in the absence of any commercial or financial relationships that could be construed as a potential conflict of interest.

## References

[1] Campbell OMR Graham WJ. Strategies for reducing maternal mortality: getting on with what works. Lancet. (2006) 368:1284–99. 10.1016/S0140-6736(06)69381-1 17027735

[2] Chen XK Wen SW Yang Q Walker MC. Adequacy of prenatal care and neonatal mortality in infants born to mothers with and without antenatal high-risk conditions. Aust NZ J Obst Gyn. (2007) 47:122–7. 10.1111/j.1479-828X.2007.00697.x 17355301

[3] Hohmann-Marriott B. The couple context of pregnancy and its effects on prenatal care and birth outcomes. Mat Child Health J. (2009) 13:745–54. 10.1007/s10995-009-0467-0 19381792

[4] Van Dijk JAW Anderko L Stetzer F. The impact of prenatal care coordination on birth outcomes. J Obst Gyn Neon Nurs. (2011) 40:98–108. 10.1111/j.1552-6909.2010.01206.x 21121950

[5] Partridge S Balayla J Holcroft CA Abenhaim HA. Inadequate prenatal care utilization and risks of infant mortality and poor birth outcome: a retrospective analysis of 28,729,765 U.S. deliveries over 8 years. Am J Perinatol. (2012) 29:287–93. 10.1055/s-0032-1316439 22836820

[6] Public Health Agency of Canada. Pregnancy, Childbirth and Caring for Newborns: Advice for Mothers During COVID-19. (2020). Available online at: https://www.canada.ca/en/public-health/services/publications/diseases-conditions/pregnancy-advise-mothers.html (accessed December 15, 2020).

[7] Dayan J Creveuil C Marks MN Conroy S Herlicoviez M Dreyfus M. Prenatal depression, prenatal anxiety, and spontaneous preterm birth: a prospective cohort study among women with early and regular care. Psychosom Med. (2006) 68:938–46. 10.1097/01.psy.0000244025.20549.bd 17079701

[8] Waldenstrom U. Experience of labor and birth in 1111 women. J Psychosom Res. (1999) 47:471–82. 10.1016/S0022-3999(99)00043-4 10624845

[9] Bergant AM Heim K Ulmer H Illmensee K. Early postnatal depressive mood: associations with obstetric and psychosocial factors. J Psychosom Res. (1999) 46:391–4. 10.1016/S0022-3999(98)00116-0 10340239

[10] Da Costa D Larouche J Dritsa M Brender W. Psychosocial correlates of prepartum and postpartum depressed mood. J Affect Disord. (2000) 59:31–40. 10.1016/S0165-0327(99)00128-7 10814768

[11] Alipour Z Lamyian M Hajizadeh E. Anxiety and fear of child-birth as predictors of postnatal depression in nulliparous women. Women Birth. (2012) 25:e37–43. 10.1016/j.wombi.2011.09.002 21959041

[12] Ngai FW Ngu SF. Predictors of maternal and paternal depressive symptoms at postpartum. J Psychosom Res. (2015) 78:155–61. 10.1016/j.jpsychores.2014.12.003 25524435

[13] Cornish AM McMahon C Ungerer JA. Postnatal depression and the quality of mother-child interactions during the second year of life. Aust J Psychol. (2008) 60:142–51. 10.1080/00049530701477738

[14] Rini CK Dunkel-Schetter C Wadhwa PD Sandman CA. Psychological adaptation and birth outcomes: the role of personal resources, stress, and sociocultural context in pregnancy. Health Psychol. (1999) 18:333–45. 10.1037//0278-6133.18.4.333 10431934

[15] Huizink AC Robles de Medina PG Mulder EJH Visser GHA Buitelaar JK. Stress during pregnancy is associated with developmental outcome in infancy. J Child Psychol Psychiatry. (2003) 44:810–8. 10.1111/1469-7610.00166 12959490

[16] Lobel M Cannella DL Graham JE DeVincent C Schneider J Meyer BA. Pregnancy-specific stress, prenatal health behaviors, and birth outcomes. Health Psychol. (2008) 27:604–15. 10.1037/a0013242 18823187

[17] Dunkel-Schetter C Lobel M. Pregnancy and birth outcomes: a multilevel analysis of prenatal maternal stress and birth weight. In Baum A Revenson TA Singer J. editors. Handbook of Health Psychology. Hove: Psychology Press (2012), pp. 431–63.

[18] Brockington I Macdonald E Wainscott G. Anxiety, obsessions and morbid preoccupations in pregnancy and the puerperium. Arch Wom Ment Health. (2006) 9:253–63. 10.1007/s00737-006-0134-z 16699837

[19] Phillips J Sharpe L Matthey S Charles M. Maternally focused worry. Arch Wom Ment Health. (2009) 12:409–18. 10.1007/s00737-009-0091-4 19626414

[20] Pampaka D Papatheodorou SI AlSeaidan M Al Wotayan R Wright RJ Buring JE. Depressive symptoms and comorbid problems in pregnancy - results from a population based study. J Psychosom Res. (2018) 112:53–8. 10.1016/j.jpsychores.2018.06.011 30097136

[21] Lebel C MacKinnon A Bagshawe M Tomfohr-Madsen L Giesbrecht G. Elevated depression and anxiety symptoms among pregnant individuals during the COVID-19 pandemic. J Affect Disord. (2020) 277:5–13. 10.1016/j.jad.2020.07.126 32777604 PMC7395614

[22] Davenport MH Meyer S Meah VL Strynadka MC Khurana R. Moms are not OK: COVID-19 and maternal mental health. Front Glob Womens Health. (2020) 1:1. 10.3389/fgwh.2020.00001 PMC8593957 34816146

[23] Wu Y Zhang C Liu H Duan C Li C Fan J. Perinatal depressive and anxiety symptoms of pregnant women during the coronavirus disease 2019 outbreak in China. Am J Obstet Gyn. (2020) 223:240.e1–9. 10.1016/j.ajog.2020.05.009 32437665 PMC7211756

[24] Ayaz R Hocaoglu M Gunay T Yardimci O Turgut A Karateke A. Anxiety and depression symptoms in the same pregnant women before and during the COVID-19 pandemic. J Perinatal Med. (2020) 48:965–70. 10.1515/jpm-2020-0380 32887191

[25] Durankus F Aksu E. Effects of the COVID-19 pandemic on anxiety and depressive symptoms in pregnant women: a preliminary study. J Matern Fetal Neonat Med. (2020) 18:1–7. 10.1080/14767058.2020.1763946 32419558

[26] Giesbrecht G. Protocol for the Canadian Pregnancy During the COVID-19 Pandemic Study. (2020). Available online at: https://psyarxiv.com/w8hd5/ (accessed December 19, 2020).

[27] Cox JL Holden JM Sagovsky R. Detection of postnatal depression - development of the 10-item Edinburgh Postnatal Depression Scale. Brit J Psychiat. (1987) 150:782–6. 10.1192/bjp.150.6.782 3651732

[28] Kozinszky Z Dudas RB. Validation studies of the Edinburgh Postnatal Depression Scale for the antenatal period. J Affect Disord. (2015) 176:95–105. 10.1016/j.jad.2015.01.044 25704562

[29] Bergink V Kooistra L Lambregtse-van den Berg MP Wijnen H Bunevicius R van Baar A. Validation of the Edinburgh depression scale during pregnancy. J Psychosom Res. (2011) 70:385–9. 10.1016/j.jpsychores.2010.07.008 21414460

[30] Cella D Riley W Stone A Rothrock N Reeve B Yount S. The Patient-Reported Outcomes Measurement Information System (PROMIS) developed and tested its first wave of adult self-reported health outcome item banks: 2005–2008. J Clin Epidemiol. (2010) 63:1179–94. 10.1016/j.jclinepi.2010.04.011 20685078 PMC2965562

[31] Reymond C Derguy C Wendland J Loyal D. French validation of a pregnancy-specific anxiety scale (PRAQ-R2). Psychol Pract. (2020) 26:231–40. 10.1016/j.prps.2018.11.008

[32] Urizar GG Jr Yim IS Rodriguez A Schetter CD. The SMART moms program: a randomized trial of the impact of stress management on perceived stress and cortisol in low-income pregnant women. Psychoneuroendocrinology. (2019) 104:174–84. 10.1016/j.psyneuen.2019.02.022 30852278

[33] Tomfohr-Madsen LM Racine N Giesbrecht GF Lebel C Madigan S. Depression and anxiety in pregnancy during COVID-19: a rapid review and meta-analysis. Psychiatry Res. (2021) 300:113912. 10.31234/osf.io/n8b7x 33836471 PMC9755112

[34] Dennis CL Falah-Hassani K Shiri R. Prevalence of antenatal and postnatal anxiety: systematic review and meta-analysis. Br J Psychiatry. (2015) 210:315–23. 10.1192/bjp.bp.116.187179 28302701

[35] Gavin NI Gaynes BN Lohr KN Meltzer-Brody S Gartlehner G Swinson T. Perinatal depression: a systematic review of prevalence and incidence. Obstet Gynecol. (2005) 106:8–14. 10.1097/01.AOG.0000183597.31630.db 16260528

[36] Lancaster CA Gold KJ Flynn HA Yoo H Marcus SM Davis MM. Risk factors for depressive symptoms during pregnancy: a systematic review. Am J Obstet Gyn. (2010) 202:5–14. 10.1016/j.ajog.2009.09.007 20096252 PMC2919747

[37] Goletzke J Kocalevent RD Hansen G Rose M Becher H Hecher K. Prenatal stress perception and coping strategies: insights from a longitudinal prospective pregnancy cohort. J Psychosom Res. (2017) 102:8–14. 10.1016/j.jpsychores.2017.09.002 28992901

[38] Dayan J Creveuil C Herlicoviez M Herbel C Baranger E Savoye C. Role of anxiety and depression in the onset of spontaneous preterm labor. Am J Epidemiol. (2002) 155:293–301. 10.1093/aje/155.4.293 11836191

[39] Simon RM Johnson KM Liddell J. Amount, source, and quality of support as predictors of women's birth evaluations. Birth. (2016) 43:226–32. 10.1111/birt.12227 26991407

[40] Madden N Emeruwa UN Friedman AM Aubey JJ Aziz A Baptiste CD. Telehealth uptake into prenatal care and provider attitudes during the COVID-19 pandemic in New York City: a quantitative and qualitative analysis. Am J Perinatol. (2020) 37:1005–14. 10.1055/s-0040-1712939 32516816 PMC7416212

[41] Hollander JE Carr BG. Virtually perfect? Telemedicine for COVID-19. N Engl J Med. (2020) 382:1679–81. 10.1056/NEJMp2003539 32160451

[42] Statistics from Statistics Canada 2016 Census of Canada. Statistics Canada. (2016).

[43] McRae L O'Donnell S Loukine L Rancourt N Pelletier C. Report summary - mood and anxiety disorders in Canada, 2016. Health Promot Chronic Dis Prev Can. (2016) 36:314–5. 10.24095/hpcdp.36.12.05 27977086 PMC5387798

[44] Pelletier L O'Donnell S McRae L Grenier J. The burden of generalized anxiety disorder in Canada. Health Promot Chronic Dis Prev Can. (2017) 37:54–62. 10.24095/hpcdp.37.2.04 28273041 PMC5607526

